# Use of Virtual Reality Tools for Vestibular Disorders Rehabilitation: A Comprehensive Analysis

**DOI:** 10.1155/2015/916735

**Published:** 2015-04-30

**Authors:** Mathieu Bergeron, Catherine L. Lortie, Matthieu J. Guitton

**Affiliations:** ^1^Department of Oto-Rhino-Laryngology and Ophthalmology, Faculty of Medicine, Laval University, Quebec City, QC, Canada G1V 0A6; ^2^Institut Universitaire en Santé Mentale de Québec, Quebec City, QC, Canada G1J 2G3

## Abstract

Classical peripheral vestibular disorders rehabilitation is a long and costly process. While virtual reality settings have been repeatedly suggested to represent possible tools to help the rehabilitation process, no systematic study had been conducted so far. We systematically reviewed the current literature to analyze the published protocols documenting the use of virtual reality settings for peripheral vestibular disorders rehabilitation. There is an important diversity of settings and protocols involving virtual reality settings for the treatment of this pathology. Evaluation of the symptoms is often not standardized. However, our results unveil a clear effect of virtual reality settings-based rehabilitation of the patients' symptoms, assessed by objectives tools such as the DHI (mean decrease of 27 points), changing symptoms handicap perception from moderate to mild impact on life. Furthermore, we detected a relationship between the duration of the exposure to virtual reality environments and the magnitude of the therapeutic effects, suggesting that virtual reality treatments should last at least 150 minutes of cumulated exposure to ensure positive outcomes. Virtual reality offers a pleasant and safe environment for the patient. Future studies should standardize evaluation tools, document putative side effects further, compare virtual reality to conventional physical therapy, and evaluate economical costs/benefits of such strategies.

## 1. Introduction

“Vertigo,” symptoms of body balance disorders of vestibular origins, such as benign paroxysmal positional vertigo (BPPV) or Ménière's disease associated vertigo, has a lifetime prevalence of 7.4% [1, 2]. As 80% of sufferers consult for their vertigo, often resulting in work interruptions, peripheral vestibular disorders represent an important cost for society [1, 3]. Most of vertigo-related expenses are due to unnecessary diagnostic measures and ineffective treatments, for example, in the case of BPPV [3].

The classical therapeutic approach for vestibular disorders relies on vestibular rehabilitation and symptomatic medication [4, 5]. Vestibular rehabilitation uses central mechanisms of neuroplasticity (adaptation, habituation, and substitution) to increase static and dynamic postural stability and to improve visuovestibular interactions in situations that generate conflicting sensory information [2, 4, 6]. Vestibular rehabilitation can improve static and dynamic balance and gait, reduce symptoms of dizziness of comorbid depression and of anxiety, and ultimately result in an increase of self-confidence and quality of life of sufferers [7].

However, many factors may negatively affect the outcome of vestibular rehabilitation, including incorrect performance of exercises and the necessity of active efforts and interest from the patient [4, 8]. Due to the variability of patients' response to therapy, there is only moderate evidence to support that vestibular rehabilitation enables symptoms recovery and improves functioning in the medium term for unilateral peripheral vestibular dysfunction [9]. Thus, more efficient and cost-effective therapeutic tools are yet to come for vestibular rehabilitation. In this context, virtual reality-based treatment could represent an interesting potential candidate.

Virtual environments are interactive simulations of real world generated by computers and presented to users through media of varying degrees of complexity (e.g., computer screen, 360° circular screen, head-mounted display, etc.). Hardware devices can be added to the equipment in order to monitor movement kinematics or provide simulations of force and haptic feedback to participants [10–13]. Given that motor skills can be learned in a virtual environment and later on applied into the real world and that virtual settings can provide controlled and/or augmented feedback on motor performance, it is not surprising that medical rehabilitation began to use heavily such settings as therapeutic tools [10]. For instance, virtual settings have been used for rehabilitation of upper extremities motor control [14–17], gaits and lower extremities control [18, 19], spatial and perceptual motor training [20–22], or balance training [23].

Although the suitability of virtual reality in balance training of participant with vestibular disorders has already been demonstrated [24], neither homogeneous data on the optimal conditions to perform virtual reality-based vestibular rehabilitation therapy nor general recommendations are currently available. Therefore, we reviewed the existing literature on virtual reality and vestibular rehabilitation to fill this gap, document this particular form of technology-enhanced medical practice, and propose recommendations for future studies and clinical applications of virtual reality tools for vestibular disorders.

## 2. Materials and Methods

A comprehensive analysis was conducted to compare the efficiency of virtual reality-based rehabilitation on peripheral vestibular disorders. Following a PICOS standardized format, this study investigates patient > 18 years with old peripheral vestibular disorders (population) in the context of virtual reality rehabilitation (intervention). We compared the impact of peripheral vestibular symptoms using validated vestibular disorder questionnaires. This was performed for different virtual reality designs and protocols (comparison), quantifying the clinical improvement of dizziness (outcome). Results of individual studies were combined under the form of a meta-analysis to quantify the improvement of vestibular disorder with virtual reality (study design).

### 2.1. Selection of Relevant Primary Studies

Studies—papers published in peer-reviewed journals excluding conference abstracts—using virtual reality-based settings for vestibular disorders rehabilitation were gathered according to the following strategy. A comprehensive search was conducted in MedLine with the following keywords: “vestibular system,” “vestibular dysfunction,” “vertigo,” “equilibrium,” “balance,” “virtual reality,” “virtual treatment,” and “virtual rehabilitation.” Keywords “treatment” or “rehabilitation” were parts of all keyword strategy to optimize the search. Articles in English, French, Spanish, and Portuguese published from 1946 to August 2013 were included in the search. The search was independently performed twice, and references were cross-checked. In addition, references from all identified studies were systematically looked for to find any supplementary sources and ensure that all relevant studies were selected. Titles and abstracts of the 489 articles identified by the search were assessed for pertinence. Of the 489 articles found on MedLine, 316 articles were duplicates and 143 articles were ignored, mainly because they did not assess our primary subject (no rehabilitation and/or no virtual therapy and/or no vestibular disorder). Studies dealing with rehabilitation on geriatric population only were also excluded, since many confounding factors may be involved in balance disorders in this particular population. Articles about a one-time diagnostic test using virtual reality without a rehabilitation or treatment process were also excluded (12 articles). Finally, we also excluded studies on vertigo of central, neurological, and/or psychiatric origin (13 articles). Three more articles were excluded for not presenting their results or having incomplete results. An initial sample of 5 articles was thus selected for further analyses from the MedLine database. The exact same procedures using an identical selection strategy were used on Google Scholar and on the Cochrane Central Register of Controlled Trials databases. These subsequent searches provided 2 supplementary articles. Therefore, the final sample was composed of 7 studies.

### 2.2. Data Extraction

Data from studies meeting inclusion criteria were extracted into a standardized database and cross-checked for accuracy. Information regarding the type of balance disorder and age of the patient was extracted from the text and the tables or obtained from the figures.

When possible, three measures of efficiency were extracted from the studies. First, the percentage of improvement on the DHI (score after rehabilitation, baseline) after the rehabilitation compared to the baseline on the Dizziness Handicap Index (DHI). Second, the percentage of improvement on another scale used by the authors (e.g., Tinetti questionnaire, Vertigo Analytic Scale; score after rehabilitation, baseline). In case of multiple scales other than the DHI, the most standardized one was favored. Third and last, the average efficiency (mean improvement of the DHI and the other scale used) was computed in order to provide a more global evaluation of the improvements and to attenuate the potential differences between the assessment tools used across the different studies.

The nature of the device used to deliver the virtual reality exposure to patients was recorded (screen in front/around the patient, goggles, head-mounted display,…) together with the potential addition of a force platform or a treadmill. Studies were further divided into either “passive” or “active” in terms of virtual reality-based rehabilitation. Passive rehabilitation required only eyes or head movements or staying immobile during the treatment. In contrast, active rehabilitation requested complete motions of the body or muscle groups in order to perform demanding movements (walking on a treadmill, doing steps, or yoga).

When available, tolerability of the rehabilitation and side effects were noted. Finally, the level of validity of each study was assessed with the Oxford grading scale [25]. The Oxford grading scale rating could range from a score of 0 (bad) to a score of 5 (good). Randomization gives a maximum of 2 points: 1 if randomization is mentioned and an additional point if the method of randomization is appropriate (1 point is deduced if inappropriate). A maximum of 2 points is given for blinding: 1 if blinding is mentioned and one additional point if the method is appropriate (1 point is deduced if inappropriate). Finally, 1 point is given if withdrawal and drop-out are described for all patients.

### 2.3. Statistical Analysis

Spearman rank tests were performed to observe whether total exposure time to virtual therapy and the number of treatment sessions were associated with the efficiency of the rehabilitation process, as measured by the DHI, other scales, and the average efficiency. Studies were also subdivided into either “low efficiency” (<20% of improvement on average efficiency) or “high efficiency” (>20% of improvement). This threshold of 20% represents in most dizziness assessment tools an improvement equivalent to a change of handicap category (no handicap/mild handicap/moderate handicap/severe handicap). Nonparametric Mann-Whitney *U* tests were then used to compare the average efficiency, total exposure time, and the number of sessions between “low efficiency” and “high efficiency” studies as well as between studies using “active” versus “passive” virtual reality settings. When normality test successfully passed, Student's *t*-tests were used. The average efficiency was analyzed as a function of total exposure time and the number of sessions using simple linear regressions.

## 3. Results

### 3.1. Characteristics of the Selected Studies

Seven studies fulfilled our criteria (Table 1), for a total of 176 subjects (including 115 patients) who underwent a protocol of virtual reality-based vestibular rehabilitation. The studies included 8 to 71 subjects with patient groups formed by 8 to 37 individuals (see the paragraph “Reliability of the Studies” for the ratio of studies having a control group). Age of the subjects ranged from 18 to 84 years. Other demographic data were impossible to extract from the studies analyzed due to a lack of information in the texts. All the following data presented here rely only on patients suffering from vestibular disorders who underwent virtual reality rehabilitation. Subjects were exposed to 6 to 12 sessions of virtual reality rehabilitation, spread over 1 to 8 weeks. Each session lasted 24 to 45 minutes, for a total of 144 to 540 minutes spent in virtual reality-based rehabilitation. Two main categories of devices were used to expose subjects to virtual reality: either screen/projection or headset/goggles. Out of the 7 studies, 5 used goggles or a head-mounted device (71%), while the other 2 used screens in front of or around the subject (28%). In addition, in the vast majority of studies (5 out of 7, 71%), a treadmill or a force platform was added to enhance the rehabilitation process.

### 3.2. Impact of Interactive Involvement

Efficacy of the virtual rehabilitation was compared regarding the type of device used. Average efficiency varied from 4.65% to 43.5% for goggles/headset and from 4.4% to 42.61% for screen/projection. The type of device used did not have an effect on efficiency (*U* tests, *P* = 0.86 for average efficiency, *P* = 0.53 for DHI evaluations and *P* = 0.86 for other scales). Efficacy of the virtual rehabilitation was also compared regarding active versus passive settings. Four studies (57%) used a passive approach, while 3 studies (43%) used a more active setting. However, no difference was observed between passive and active settings on average efficiency (*U* test, *P* = 0.63), DHI scores (*U* test, *P* = 0.53) and other scales scores (*U* test, *P* = 0.23).

### 3.3. Efficiency and Impact of Treatment Duration

A clear improvement following virtual reality-based therapy was observed in all studies whatever the assessment tool used. After completing all sessions, the average efficiency across studies varied between 4.4 to 43.5% (DHI score (4–63%) and other scales (4.4–51%)). In studies using the DHI, the mean decrease was 26 points over 100 on the scale, allowing patients to lessen their handicap either from severe to moderate or from moderate to mild.

**Figure 1:**
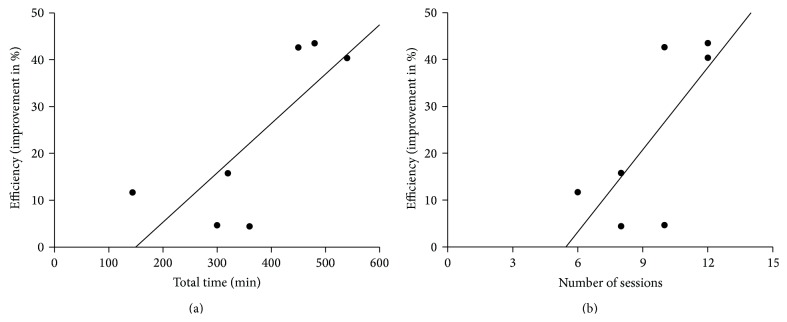
Impact of the duration of virtual reality exposure on treatment efficiency. (a) Average efficiency in terms of symptoms' improvement as a function of the total time spent in virtual reality-based therapy (linear regression, *r* ^2^ = 0.5975, *P* < 0.05). (b) Average efficiency in terms of symptoms' improvement as a function of the total number of sessions of virtual reality-based treatment (*r* ^2^ = 0.5164, *P* = 0.07).

Despite statistical trends, efficiency was not directly associated neither with total exposure time in virtual reality-based rehabilitation (Spearman rank tests, *P* = 0.24 for DHI evaluation, *P* = 0.09 for other scales and for average efficiency) nor with the number of sessions (Spearman rank tests, *P* = 0.36 for DHI evaluation, *P* = 0.09 for other scales and for average efficiency). Time per session was not related to efficiency either (Spearman rank tests, *P* = 0.17 for DHI evaluation, *P* = 0.78 for other scales and *P* = 0.60 for average efficiency). However, simple linear regressions revealed that the average efficiency was significantly explained by total exposure time (*r* ^2^ = 0.5975, *P* < 0.05, Figure 1(a)) and in a lesser extent by the number of sessions (*r* ^2^ = 0.5164, *P* = 0.07, Figure 1(b)).

**Figure 2:**
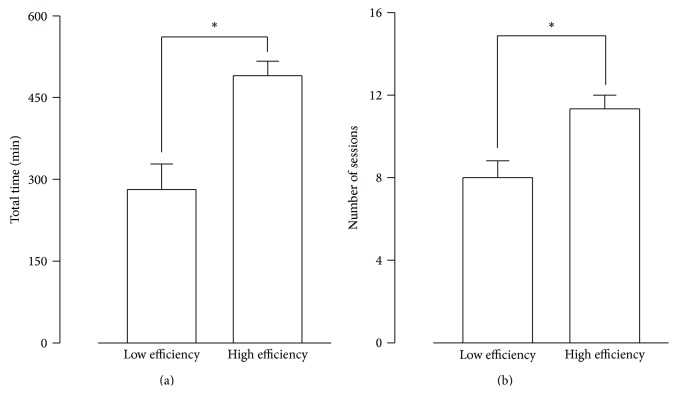
Differential characteristics of virtual reality protocols according to average efficiency. (a) Time spent in virtual reality-based treatment. (b) Number of sessions depending on the clinical impact of the treatment (“low efficiency” defining studies with less than 20% of improvement on average efficiency and “high efficiency” studies with more than 20% of improvement). ^∗^ *P* < 0.05.

As stated earlier, studies were divided into either “low efficiency” (<20% of improvement on average efficiency) or “high efficiency” (>20% of improvement, *t*-test, *P* < 0.001), (Table 2). Studies with lower efficiency had shorter total time spent in rehabilitation (*t*-test, *P* < 0.05, Figure 2(a)) and fewer sessions than more efficient studies (*t*-test, *P* < 0.05, Figure 2(b)).

### 3.4. Occurrence of Side Effects

Surprisingly, although a majority of studies mentioned that the rehabilitation was well tolerated, the side effects were almost never documented. Particularly, none of the studies evaluated motion sickness and/or cybersickness with a validated questionnaire. When mentioned, side effects and tolerability were only briefly described (5 of the 7 studies). In terms of tolerability, no study reported major issues following the use of virtual reality and no significant incident or fall was reported.

### 3.5. Reliability of the Studies

Only 4 out of the 7 studies had a control group, with only one study randomized (Table 3). Ratings using the Oxford grading scale [25] to evaluate the methodological quality of clinical trials revealed significant weaknesses. The studies ranged from 1 (3 studies out of 7; 43%) to 2 (3 studies out of 7; 43%), with only one study reaching a score of 3 out of a maximum score of 5 (14%)—the minimum score for a study to be considered as acceptable.

## 4. Discussion

This comprehensive analysis confirmed that the utilization of virtual reality in the context of vestibular disorders could be a very valuable approach. Indeed, an improvement of the patients' symptoms has been documented in all the studies examined. With an average evaluation of the vertigo-related handicap going from moderate to mild at the end of the virtual reality-based rehabilitation, these emerging tools should not be neglected among the therapeutic arsenal when dealing with patients suffering from vestibular disorders. However, despite these promising results, further research is needed to document the exact parameters of an optimal protocol and to define the most cost-effective strategies.

### 4.1. Protocol Design and Assessment

In the context of defining an evidence-based strategy of virtual reality-based therapies for vestibular disorders, the relative methodological weakness of the studies examined was a major issue. Indeed, none of the selected studies ranked more than 3 on the Oxford grading scale. More worrying, the majority of studies ranked 1 or 2 (i.e., low methodological quality). These low ratings were mostly due to the absence of control groups, randomized conditions, and blind experiments. However, it should be noted here that, due to the nature of the diseases and of the rehabilitation interventions, blinding of the protocols is almost impossible to reach, partly explaining the relatively low score observed. Unfortunately, the small size of most of the cohorts combined with nonsystematic evaluations made it also difficult to reach absolute conclusions.

While increasing the group sizes or multiplying control groups could be difficult to do in the context of costly experiments involving patients and important resources, efforts should be done regarding the rigorousness and standardization of evaluation. For instance, the DHI was not systematically used. While many scales or questionnaires could be used to document vestibular disorder-related handicaps, the DHI still remains one of the most standard and easy to administer assessment tools [26]. A few studies preferred using nonvalidated “homemade” questionnaires. These questionnaires do not allow a direct and standardized comparison with the literature. Thus, they should be avoided or used only in conjunction with validated tools such as the DHI.

### 4.2. Practical Optimization

In a practical point of view, a very important issue is which of time spent in virtual reality-based training and the number of session is the key factor to increase the therapeutic effect. Interestingly, the present meta-analysis seemed to suggest that time spent in virtual reality-based therapy contributed more to the average efficiency than the number of sessions. Results unveil that a minimum exposure time of 120–150 minutes is required to detect a quantifiable benefit for the patient. However, the time spent and the number of sessions are intimately related. Furthermore, the effect of intertrial time (time between two consecutive sessions) has been so far overlooked. This parameter should be documented in future studies.

Given that time spent in virtual therapy is clearly of importance, longer sessions in a short period of time could be effective and convenient. However, the total duration of a session is strongly depending on the physical state of the patient. Of note, some of the results of this study might appear contradictory. However, that might be explained by the very size of our sample (only 7 studies met our criterions), limiting the overall power of a few analyses.

### 4.3. Clinical Applicability

Peripheral vestibular disorders can be heterogeneous in terms of etiology [27]. One of the strengths of this meta-analysis is that the studies examined gathered diverse populations of patients presenting various peripheral vestibular disorders similar to ones found in clinical settings.

Virtual reality settings are extremely useful for various pathologies [14]. However, one of the main limitations of using such protocols as clinical tools is the related cost. In the context of important attempts in cost reductions in health care systems, this issue could be a major argument against the implementation of virtual reality settings in clinical facilities. Although more studies have to be dedicated to answer the question on cost/efficiency of virtual reality in clinical situations, the case of vestibular rehabilitation seems encouraging. Indeed, our evidence-based data suggest that there is no need for the most expensive devices to obtain significant improvement in patients. Instead, very positive outcomes can be evidenced with affordable devices such as a Nintendo Wii. Furthermore, even if we did not observe a difference in efficiency between active and passive protocols, technological devices allowing active mobilization of muscular groups can be acquired with limited cost. Self-utilization of virtual reality devices by patients could in fact reduce the rehabilitation costs.

This leads us to a second limitation. None of the studies analyzed answered the question of whether virtual reality-based vestibular rehabilitation should be done alone or in combination with conventional vestibular rehabilitation. Intuitively, one could expect that the combination of various therapeutic protocols would have optimal results. However, this has to be demonstrated.

Another consideration limiting the use of virtual reality-based settings in rehabilitation medicine is cybersickness. Indeed, due to unnatural and sometimes conflicting multisensory stimuli, exposure to interactive virtual environments can cause discomfort during or after the session, which is referred to as cybersickness [28–30]. Symptoms reported are motion sickness-like, including nausea, vomiting, headache, somnolence, loss of balance, and altered eye-hand coordination [29]. These undesirable events, which can be distinguished from classical motion sickness caused by vestibular stimulation alone, are particularly worrying in participants with impaired vestibular function. While, to date, most studies have underlooked this issue, the occurrence of cybersickness should be systematically documented before virtual rehabilitation could be used on larger scales for these populations of patients. However, despite these limits, the absence of reported side effects or adverse events (e.g., falls) so far tends to support the notion that virtual rehabilitation is well tolerated and could be safely used in a rehabilitation setting.

### 4.4. Conclusions and Recommendations

The present meta-analysis demonstrates the promising potential of virtual reality-based treatment for peripheral vestibular disorders. Despite significant differences in terms of protocol used and outcomes evaluation, all studies demonstrated that virtual reality-based rehabilitation strategies had a positive effect and were seemingly well tolerated. The main criterion predicting treatment success and magnitude of symptoms improvement is the total time spent in virtual reality training. The complexity of the setting used does not seem to have a direct impact on efficiency, as important results are possible with inexpensive settings. Thus, virtual reality-based rehabilitation represents a potentially promising new avenue to reduce the costs of peripheral vestibular disorders rehabilitation.

In conclusion, some recommendations are proposed for future studies to standardize intervention protocols and evaluation tools, document side effects, determine if virtual reality-based rehabilitation should be combined with classical rehabilitation, and define profiles of patients susceptible to benefit from a virtual reality-based rehabilitation as follows.

*Recommendations for Virtual Reality-Based Treatment Applied to Peripheral Vestibular Disorders*

- Use only validated assessment tool, including the DHI as primary assessing tool.
- Document clearly the time and number of sessions spent in rehabilitation and time between sessions.
- Document virtual reality-related side effects (cybersickness) with validated questionnaire, such as Simulator Sickness Questionnaire (SSQ).
- Document complications of virtual reality rehabilitation such as falls and fractures.
- Document symptomatic medication taken by the patient.
- If possible, document the cost of the device and each session.

## Figures and Tables

**Table 1:** Main characteristics of the studies.

Study	Patients/ages	Vestibular problem	Type of virtual reality device	Measurements of efficacity

dos Santos et al. (2009) ACTA ORL [31]	*n* = 8 (18–60 years, mean 41 years)	Chronic vestibular dysfunction	Balance Rehabilitation Unit (BRU) with virtual reality glasses projecting visual stimuli	(i) Dizziness Handicap Index (DHI) (ii) Vertigo analogic Scale (VAS) (iii) Stabilisation limits (LOS) (iv) Audiometer (tonal, vocal) (v) Impedance (vi) Vectonystagmography (vii) Posturography

Rodrigues et al. (2009) Rev Equilibro Corporal e Saude [32]	*n* = 10 (24–76 years, mean 51 years)	Chronic vestibular disorder secondary to Ménière's disease	Balance Rehabilitation Unit (BRU) with visual stimuli (glasses)	(i) DHI (ii) VAS (iii) LOS (iv) Computerized Posturography

Viirre and Sitarz (2002) Laryngoscope [33]	*n* = 15 (age n/a) (i) *n* = 9 patients (ii) *n* = 6 controls	Vertigo symptoms for more than 6 months (with no improvement for at least 6 months)	Head-mounted Display (HMD) much like a visor with mounted video screens	(i) DHI (ii) Vestibuloocular reflex (VOR)

Pavlou et al. (2012) J Vestib Res [34]	*n* = 16 (18–75 years, mean 40 years) (i) *n* = 11 (Group S = static virtual reality) (ii) *n* = 5 (Group D = dynamic virtual reality) (iii) *n* = 5 Group D1 (5 patients from Group S who had also dynamic treatment)	Confirmed peripheral vestibular deficit (caloric test and/or rotational test on ENG)	ReaCtoR in the Department of Computer Science: immersive projection theatre (IPT). 3 rear-projected vertical screens (3 m × 2.2 m)	(i) Situational Vertigo Questionnaire (ii) Beck Depression Inventory (iii) Beck Anxiety Inventory (iv) Fear Questionnaire (v) Dynamic Gait Index (DGI) (vi) Virtual reality exercise symptom scores (VRCESS)

Sparrer et al. (2013) Acta Otolaryngol [35]	*n* = 71 (28–84 years, mean 43 years) (i) *n* = 37 patients (ii) *n* = 34 controls	Acute vestibular neuritis (sudden, spontaneous, and unilateral loss of peripheral vestibular function within 48 h of the onset of vertigo)	Wii Fit balance board with image on screen	(i) DHI (ii) Wii Fit age (iii) Sensory Organization Test (SOT) (iv) Vertigo Symptom Scale (VSS) (v) Tinetti questionnaire

Whitney et al. (2009) Physical Therapy Reviews [36]	*n* = 12 (18–80 years, mean 52 years)	Vestibular disorders with dizziness and loss of balance	Treadmill in a virtual grocery store on a screen	(i) DHI (ii) Activities-specific Balance Confidence Scale (ABC) (iii) Dynamic Gait Index (DGI) (iv) Timed Up and Go (TUG) (v) Sensory Organization Test (SOT)

Garcia et al. (2013) Braz J Otorhinolaryngol [37]	*n* = 44 (18–60 years, mean 48 years) (i) *n* = 23 cases (ii) *n* = 21 controls	Unilateral or Bilateral Ménière's disease	Balance Rehabilitation Unit (BRU) with virtual reality glasses projecting visual stimuli	(i) DHI (ii) Analog dizziness scale (iii) ENT examination (iv) PTA audiometry, impedance (v) Functional vestibular examination (vi) Speech intelligibility testing (vii) Posturography

**Table 2:** Efficiency of rehabilitation regarding the type of device.

Study	Efficiency	Active versus passive	Average efficiency
dos Santos et al. (2009) [31] ACTA ORL	Low	Passive	15.75%

Rodrigues et al. (2009) [32] Equilíbrio Corporal e Saúde	High	Passive	43.50%

Viirre and Sitarz (2002) [33] Laryngoscope	Low	Passive	4.65%

Pavlou et al. (2012) [34] J Vestib Res	Low	Active	4.40%

Sparrer et al. (2013) [35] Acta Otolaryngol	High	Active	42.61%

Whitney et al. (2009) [36] Physical Therapy Reviews	Low	Active	11.67%

Garcia et al. (2013) [37] Braz J Otorhinolaryngol	High	Passive	40.35%

**Table 3:** Studies reliability assessed according to the Oxford grading scale.

Study	Oxford scale	Control group	Limitations
dos Santos et al. (2009) [31] ACTA ORL	1	N	Limited number of patients

Rodrigues et al. (2009) [32] Equilíbrio Corporal e Saúde	1	N	Limited number of patients No control

Viirre and Sitarz (2002) [33] Laryngoscope	2	Y	Limited number of patients Limited demographic data

Pavlou et al. (2012) [34] J Vestib Res	2	Y	Unique and specific virtual reality device

Sparrer et al. (2013) [35] Acta Otolaryngol	2	Y	Limited number of patients

Whitney et al. (2009) [36] Physical Therapy Reviews	1	N	Limited number of patients No control

Garcia et al. (2013) [37] Braz J Otorhinolaryngol	3	Y	Patients also on medication (betahistine)
